# Autoimmune Thyroid Diseases After COVID-19 Infection: A Systematic Review of Clinical Manifestation and Outcomes

**DOI:** 10.3390/ijerph23060689

**Published:** 2026-05-22

**Authors:** Assylzhan M. Messova, Ilvira Ganiyeva, Sagira T. Abdrakhmanova, Aliya Tuleubayeva, Makhmutbay Sanbayev, Makpal G. Makibayeva, Amin Tamadon

**Affiliations:** 1 Pediatric Diseases Department, Astana Medical University, Astana 010000, Kazakhstan; messova.a@amu.kz (A.M.M.); ilvira_ganieva@bk.ru (I.G.); abdrakhmanova.s@amu.kz (S.T.A.); makibaeva.m@amu.kz (M.G.M.); 2 Department of Traumatology and Pediatric Surgery, Semey Medical University, Semey 071400, Kazakhstan; 3 Department of Natural Sciences, West Kazakhstan Marat Ospanov Medical University, Aktobe 030000, Kazakhstan; amintamaddon@yahoo.com

**Keywords:** COVID-19, SARS-CoV-2, autoimmune thyroid disease, Graves’ disease, Hashimoto’s thyroiditis, thyroid autoimmunity, post-COVID-19 complications

## Abstract

**Highlights:**

**Public health relevance—How does this work relate to a public health issue?**

- SARS-CoV-2 is increasingly recognized as a trigger for autoimmune thyroid diseases (AITDs), including Graves’ disease and Hashimoto’s thyroiditis—common disorders disproportionately affecting women of reproductive age—making this a direct post-pandemic public health concern.
- Evidence documents rising thyroid autoantibody prevalence after infection with COVID-19, with risk amplified by disease severity, and rare life-threatening complications (thyroid storm, myxedema coma, and dilated cardiomyopathy). This highlights the need for timely recognition and multidisciplinary management.

**Public health significance—Why is this work of significance to public health?**

- This systematic review (2020–2025) synthesizes 46 geographically diverse studies, providing clinicians and public health professionals with a consolidated, evidence-based resource for recognizing and managing AITD after a COVID-19 diagnosis.
- Graves’ disease presents with more aggressive thyrotoxicosis post-COVID-19 than non-viral cases. Its overlap of symptoms with Long COVID complicates diagnosis, underscoring the need for systematic thyroid function assessments in post-COVID-19 follow-up pathways.

**Public health implications—What are the key implications or messages for practitioners, policy makers, and/or researchers in public health?**

- Clinicians should consider conducting thyroid function testing (TSH, fT3, fT4, autoantibodies when indicated) in patients with persistent fatigue, palpitations, weight change, or neuropsychiatric symptoms after a diagnosis of COVID-19, especially women and those with autoimmune risk factors. Severe or atypical presentations require prompt multidisciplinary evaluation.
- Policy makers and research funders should prioritize prospective multicenter cohort studies, long-term follow-up, and international registries with standardized reporting to understand the true incidence of AITD post-COVID-19 infection and identify high-risk groups for targeted surveillance.

**Abstract:**

**Background:** Increasing evidence suggests that COVID-19 can induce or exacerbate autoimmune disorders, including immune-mediated thyroid dysfunction. The most common autoimmune thyroid diseases are Graves’ disease and Hashimoto’s thyroiditis; the mechanisms by which viral infections like SARS-CoV-2 trigger these diseases are not fully understood. **Objectives:** This study aims to systematically review published clinical evidence on the presentation, laboratory characteristics, and outcomes of autoimmune thyroid diseases after COVID-19 infection. **Methods:** The review followed the PRISMA 2020 framework. Scopus, Web of Science, and PubMed were searched for English-language studies between January 2020 and December 2025 using the terms COVID-19, SARS-CoV-2, autoimmune thyroiditis, Graves’ disease, Hashimoto’s thyroiditis, and autoimmune thyroid disease. **Results:** In total, 46 studies (five cohort studies and 41 case reports/series) involving 3856 patients were analyzed. The findings indicate that a significant increase in TPOAb prevalence occurs post-COVID-19 infection (15.7% vs. 7.7% in controls). New-onset Graves’ disease (GD) post-COVID-19 presented with higher fT3/fT4 ratios and more aggressive thyrotoxicosis compared to non-viral cases. Rare but severe manifestations included thyrotoxic periodic paralysis, Hashimoto’s encephalopathy, and dilated cardiomyopathy. **Conclusions:** SARS-CoV-2 may act as a trigger for autoimmune thyroid diseases, particularly in moderate-to-severe infections; however, the strength of this association warrants further investigation with controlled prospective data. Standard therapy remains effective, but thyroid function monitoring is advisable during post-COVID-19 recovery. An interdisciplinary approach is essential for early diagnosis and management of systemic complications.

## 1. Introduction

The coronavirus disease 2019 (COVID-19) pandemic, caused by the severe acute respiratory syndrome coronavirus 2 (SARS-CoV-2), caused significant global morbidity and mortality. Since then, recognition of its multi-systemic sequelae has increased [1]. Beyond acute respiratory illness, SARS-CoV-2 infection has been implicated in immune dysregulation, potentially triggering or exacerbating a range of autoimmune conditions [2].

The thyroid gland represents a plausible target for such post-infectious autoimmunity. It exhibits high expression of angiotensin-converting enzyme 2 (ACE2), the primary receptor for SARS-CoV-2 viral entry [3]. Autoimmune thyroid diseases (AITDs), namely Graves’ disease and Hashimoto’s thyroiditis, are common organ-specific disorders characterized by an immune response against thyroid antigens [4]. Emerging evidence suggests that SARS-CoV-2 may precipitate AITD through several hypothesized mechanisms, including direct viral cytopathy, cytokine-mediated immune activation, and molecular mimicry between viral epitopes and thyroid proteins [5,6].

Since the onset of the COVID-19 pandemic, numerous case reports, series, and observational studies have described the de novo onset or recurrence of AITDs following COVID-19, typically within weeks of infection [7,8]. However, early prospective observational studies were limited to initial reports and a small number of cases [9]. A comprehensive, up-to-date synthesis of the expanding literature is required to clarify the clinical spectrum, diagnostic features, management, and outcomes of AITDs post-COVID-19 infection.

Since 2024, additional systematic reviews have examined related aspects of COVID-19 thyroid pathology, including overall changes in thyroid function [10] and post-COVID-19 subacute thyroiditis [11], further underscoring the need for a focused synthesis on AITD specifically.

Therefore, this systematic review aims to synthesize the current evidence (2020–2025) on autoimmune thyroid diseases following SARS-CoV-2 infection. Specifically, we will examine the demographic, clinical, laboratory, and imaging characteristics of affected patients, detail treatment modalities and responses, and summarize clinical outcomes. This work aims to provide clinicians with a consolidated evidence base to aid timely recognition and management of post-COVID-19 sequelae and highlight key areas for future research. The research question was structured using the PEO (Population, Exposure, Outcome) framework. Patients were included if they had a diagnosis of AITD, confirmed prior exposure to COVID-19, and available data on clinical, laboratory, and therapeutic outcomes.

## 2. Materials and Methods

### 2.1. Eligibility Criteria

Clinical reports contained in this review covered autoimmune thyroid disorders (AITDs) with onset or exacerbation of previously known conditions after a known COVID-19 infection. Included criteria were eligible designs (case reports, case series, and observational studies) that enrolled adults (≥18 years) with laboratory-confirmed SARS-CoV-2 infection, as determined by polymerase chain reaction or serologic testing. The literature also needed to provide diagnostic data indicating autoimmune thyroid disease (Hashimoto thyroiditis, Graves’ disease, or mixed forms) with at least one of the following: positive thyroid autoantibodies (TPOAb, TgAb, TRAb, or TSI); typical imaging (thyroid ultrasound or scintigraphy); or typical biochemical patterns (abnormal TSH and fT4/fT3 levels).

Autoantibody positivity was not a mandatory inclusion criterion, as enforcing it retrospectively would have introduced temporal bias and would not reflect routine clinical practice. Instead, diagnostic certainty was graded post hoc into two tiers. Cases were classified as high confidence when at least one thyroid autoantibody (TPOAb, TgAb, TRAb, or TSI) was positive; this applied to the majority of included studies. Cases lacking autoantibody data were classified as moderate confidence provided they satisfied two or more of the following: (a) TSH suppression below 0.01 mIU/L with fT3/fT4 elevation inconsistent with non-thyroidal illness; (b) ultrasonographic findings typical of autoimmune thyroiditis, such as diffuse hypoechogenicity, parenchymal heterogeneity, or increased vascularity without tenderness; (c) a documented therapeutic response to antithyroid drugs or levothyroxine; (d) scintigraphic findings consistent with Graves’ disease. Cases that remained indistinguishable from subacute thyroiditis were excluded. Only articles written in the English language, with full-text and peer-reviewed content published between 2020 and December 2025, were included. Abstracts of conferences that lacked sufficient data, preprints that were not peer-reviewed, reviews, editorials, commentaries, and animal studies were excluded.

The studies included were required to have extractable data on demographics, post-infection interval, clinical features, treatment strategy, and outcomes to be considered comparable. This review was restricted to adults (age ≥ 18), as autoimmune thyroid diseases behave quite differently in children—both clinically and immunologically—and mixing the two populations would obscure meaningful patterns in the data.

### 2.2. Sources of Information and Search Strategy

**Figure 1:**
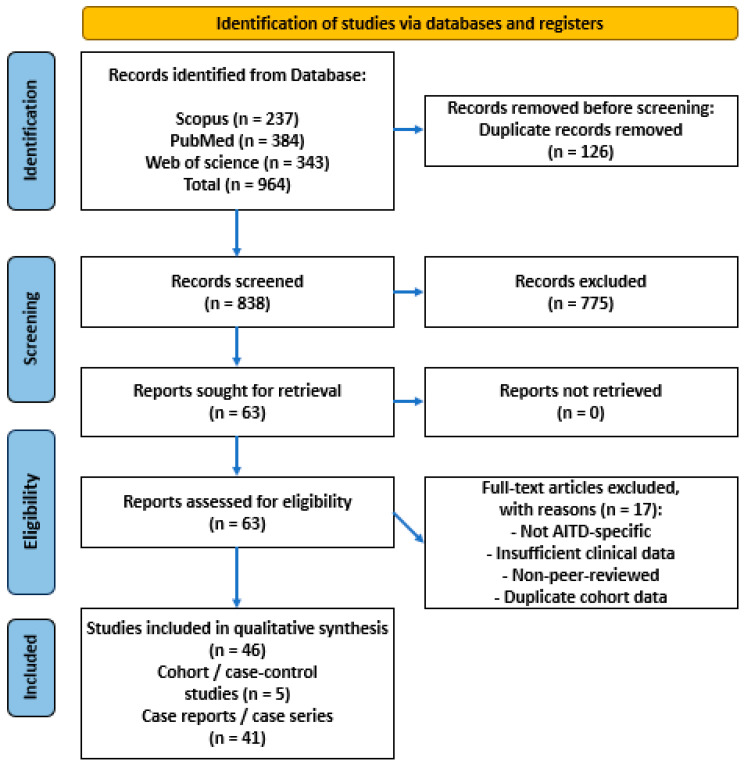
PRISMA 2020 flow diagram illustrating the study selection process for the systematic review of autoimmune thyroid diseases post-COVID-19 infection.

The research was conducted in accordance with the PRISMA 2020 guidelines [12]. A completed PRISMA 2020 checklist is provided as Supplementary Material (Table S1). The review protocol was prospectively registered with the International Prospective Register of Systematic Reviews (PROSPERO) under registration number CRD420261280025. The study selection process is illustrated in the PRISMA 2020 flow diagram (Figure 1). PubMed (*n* = 384), Scopus (*n* = 237), and Web of Science (*n* = 343) were searched. Screening of records resulted in 838 records after duplicates were removed (*n* = 126). A qualitative synthesis was conducted on 46 studies that met the inclusion criteria (confirmed cases of COVID-19 and subsequent new-onset or recurrent AITD).

The Boolean operators used were (COVID-19 OR SARS-CoV-2 OR Coronavirus) AND (autoimmune thyroiditis OR Hashimoto thyroiditis OR Graves’ disease OR autoimmune thyroid disease OR thyroid autoimmunity OR autoimmunity).

Medical Subject Headings (MeSH) were used in conjunction with free-text searches to retrieve both indexed and non-indexed records [13,14]. Eligible papers were manually screened (backward citation tracking), and forward searches in Google Scholar were conducted to identify newer, relevant reports [14,15]. All retrieved citations were copied into a spreadsheet for manual screening and deduplication. Titles and abstracts were filtered, and the full text was then checked against predetermined inclusion and exclusion criteria. The selection process is summarized in the PRISMA flowchart (Figure 1).

### 2.3. Selection Process

Two reviewers manually screened and extracted data in accordance with PRISMA 2020 guidelines [12,16]. Title and abstract screening: Irrelevant records and duplicates were excluded. Full-text review: The remaining studies were evaluated against the specified eligibility criteria. Final inclusion: Articles that fulfilled all the requirements were included in the synthesis.

### 2.4. Data Extraction and Items

Data extraction was conducted using a structured template based on the Cochrane Handbook of Systematic Reviews of Interventions [16] and the JBI Manual of Evidence Synthesis [17]. Based on each study, the following data were obtained: the author(s), year of publication, and the country where the study was performed; study design and cases; the type of autoimmune thyroid disorder; the time after COVID-19 onset; significant laboratory results (TSH, fT4, fT3, TPOAb, TgAb, and TRAb/TSI); radiographic findings; treatment options (antithyroid drugs, levothyroxine, corticosteroids, β-blockers, radioiodine, IVIG, or surgery); and clinical outcomes (remission, relapse, or persistent symptoms). Missing or unclear information was confirmed in the text or tables of every article; no imputation of data or contact with the author was carried out. Synthesis of all extracted data was performed with rechecking.

### 2.5. Risk of Bias Assessment

The Joanna Briggs Institute (JBI) Critical Appraisal Tools were used to evaluate each included study, using the case report and case series appraisal tools [18]. The authors assessed six key areas: the clarity of patient demographics, the completeness of clinical history, the validity of diagnostic confirmation, the suitability of therapeutic interventions, the adequacy of follow-up, and the plausibility of causation between COVID-19 and AITD. Studies that met ≥5 criteria were categorized as low risk, those with 3–4 criteria were categorized as moderate risk, and those with ≤2 criteria were categorized as high risk. Two reviewers assessed the internal consistency of assessments.

### 2.6. Effect Measures and Synthesis

The available evidence (primarily from single-patient reports) was descriptive; therefore, quantitative pooling or meta-analysis was not feasible [19,20,21]. In place of this, a narrative synthesis was used to summarize trends in the studies. Clinical and laboratory outcomes were categorized by AITD type (Graves’ disease, Hashimoto disease, or mixed), the interval following infection (short: <4 weeks; intermediate: 4–8 weeks; long: >8 weeks), modality of therapy, and geographic origin. Proportions and qualitative patterns were reported to be present. Studies were cross-compared at different risk-of-bias levels, and the consistency of findings was evaluated. Each analysis and tabulation was calculated using MS Excel. No statistical modeling or sensitivity analysis was conducted because the sample sizes were small and heterogeneous.

### 2.7. Protocol Registration

This systematic review was conducted in accordance with a predefined protocol registered with the International Prospective Register of Systematic Reviews (PROSPERO) under registration number CRD420261280025.

## 3. Results

### 3.1. Study Selection

The systematic search and selection process is summarized in the PRISMA flow diagram (Figure 1). A total of 964 records were identified from the databases. After removing 126 duplicates, 838 records were screened based on their titles and abstracts. Following the full-text assessment of 63 articles, 46 studies met the inclusion criteria and were included in the qualitative synthesis.

The final sample comprised five observational cohort studies and 41 case reports or case series. The included studies were geographically diverse, with contributions from North America, Europe, Asia, South America, and the Middle East [22,23,24,25,26,27,28,29,30,31,32,33,34,35,36,37,38,39,40,41,42,43,44,45,46,47,48,49,50,51,52,53,54,55,56,57,58,59,60,61,62,63,64,65,66,67,68,69]. A summary of the key characteristics, findings, and outcomes of all included studies is provided in Table 1.

The following descriptive proportions were identified for seropositive cases among the 46 studies that were included. Of those described as having an AITD phenotype, Graves’ disease accounted for 72% (33/46; 95% CI: 57–84%), Hashimoto’s thyroiditis for 17% (8/46; 95% CI: 8–31%), and mixed or transitional forms for 11% (5/46; 95% CI: 4–24%). The overall time it took from a person being diagnosed as having had SARS-CoV-2 to developing AITD was approximately 42 days after their positive diagnosis (IQR: 14–84); however, there is some variation depending on whether someone develops Graves’ disease (median of 45 days; IQR: 21–90) versus Hashimoto’s thyroiditis (median of 28 days; IQR: 14–49). Follow-up data was available for 45 of these cases, and there were reported to be clinical improvements in 43 of them (or 96 percent; 95% CI = 84–99%) following treatment. Fatalities were reported in two individuals (or 4 percent; 95% CI = 0.5–14%) as well.

Diagnostic confidence was high (antibody-confirmed) in 42 of the 46 included studies (91%) and moderate (clinically confirmed without documented autoantibody data) in the remaining four studies (9%; studies [36,49,50,66]). A sensitivity check excluding the four moderate-confidence cases did not alter the overall pattern of findings (Table 1).

### 3.2. Demographic and Temporal Profile

A strong female predominance was observed across the reported cases (~81%), aligning with the known epidemiology of AITDs. The mean age of patients ranged from 23 to 58 years. The interval between a confirmed SARS-CoV-2 infection and the onset or exacerbation of AITD symptoms ranged from several days to several months, with the majority of cases (*n* = 37) occurring within a distinct window of 2 to 8 weeks post-infection [23,25,44].

### 3.3. Laboratory and Immunological Findings

Cohort data indicated a significant increase in thyroid autoimmunity biomarkers following COVID-19. One prospective study of 599 survivors found a doubling in the prevalence of thyroid peroxidase antibodies (TPOAb) compared to controls (15.7% vs. 7.7%) [22]. Another large retrospective cohort (*n* = 2339) confirmed elevated levels of TPOAb and thyroglobulin antibodies (TgAb) during a 6-month follow-up, with levels correlating with the initial severity of COVID-19 [23].

A key differentiating feature of Graves’ disease after COVID-19 infection was found in a multicenter case–control study [25]. Patients with Graves’ disease triggered by SARS-CoV-2 presented with significantly higher free triiodothyronine (fT3) levels and fT3/free thyroxine (fT4) ratios compared to patients with Graves’ disease unrelated to viral infection, suggesting a more intense thyrotoxic state. Significantly elevated fT3 values and fT3 to fT4 ratios are consistent with a more severe thyrotoxicosis; however, no significant difference was found in terms of serum TRAb titer when comparing patients with post-COVID-19 Graves’ disease versus those with non-COVID-19 Graves’ disease. The disparity between the degree of functional severity (thyrotoxicosis), as measured by both fT3 and fT3-to-fT4 ratios, and the serologic evidence of an autoimmune response (TRAb titer) suggests that SARS-CoV-2 may have enhanced the sensitivity of TSH receptors and/or altered the responsiveness of the thyroid to TSH through mechanisms not solely involving increased production of autoantibodies to TSH receptors, but potentially via a cytokine-mediated “storm” effect or direct viral impact on the thyrocyte [25].

### 3.4. Clinical Spectrum and Disease Phenotypes

The analysis of included studies revealed a distinct distribution of autoimmune thyroid disease (AITD) phenotypes following COVID-19 infection. Graves’ disease (GD) was the most frequently reported condition, identified as the primary diagnosis in 33 of the 46 studies (72%) included in this review [25,27,28,29,30,31,32,35,36,37,38,39,40,41,43,44,45,46,47,49,50,51,53,54,56,57,58,59,60,61,62,65,66,67]. This was followed by Hashimoto’s thyroiditis (HT), reported in eight studies (17%) [24,33] (case 2), ref. [39] (case 1), refs. [42,46,48,56,63].

Several reports described mixed or transitional forms, indicative of post-COVID-19 immune dysregulation. Notably, a case series (Du et al., 2024) documented five patients with shifts in thyroid involvement, such as conversion from Hashimoto’s to Graves’ disease [34]. Another case report described the concurrent presentation of subacute thyroiditis followed by persistent Graves’ disease [65]. These phenomena underscore the potential for SARS-CoV-2 infection to trigger dynamic and overlapping autoimmune thyroid responses.

While typical symptoms of thyrotoxicosis (palpitations, tremors, weight loss) or hypothyroidism (fatigue and cold intolerance) predominated, the review identified several rare and severe manifestations expanding the clinical spectrum. The clinical manifestation varied according to the specific AITD phenotype. A case of apathetic hyperthyroidism is described—a rare variant of hyperthyroidism—which is more common in older people. Clinical symptoms (tremors, tachycardia, and sweating) were mild, and the patient complained of anorexia, weakness, jaundice, nausea, vomiting, and fatigue. This case demonstrates that individuals who have recovered from COVID-19 may develop an unusual, “latent” (apathetic) form of autoimmune hyperthyroidism, which complicates clinical diagnosis [40].

Another atypical manifestation of thyrotoxic periodic paralysis (acute flaccid quadriparesis due to thyrotoxic hypokalemic periodic paralysis) was documented [53]. The clinical spectrum was expanded to include neurological masks: thyrotoxic periodic paralysis patients who manifested with four-limb weakness, known as quadriparesis [53], Hashimoto’s encephalopathy with cognitive decline [63], and cardiomyopathy with an LVEF of 29% [67] and fatal thyroid storms [62]. New-onset or exacerbated Graves’ orbitopathy was reported in several cases [30,47,54].

Mixed forms were more likely to reflect a shift in thyroid function, supporting the notion of immune dysregulation after COVID-19 infection. The laboratory findings in Graves’ disease or Hashimoto thyroiditis presented as depressed TSH and high fT4/fT3 or high TSH and low FT4. Most patients had positive thyroid autoantibodies (TRAb, TSI, TPOAb, and TgAb). Thyroid ultrasound often showed increased vascularity, diffuse hypoechogenicity, or heterogeneous parenchymal structure [4,5].

### 3.5. Treatment and Outcomes

Treatment strategies followed established guidelines for AITDs. Hyperthyroidism was primarily managed with antithyroid drugs (methimazole/carbimazole or propylthiouracil), often in combination with beta-blockers for symptom control. For severe or complicated cases, therapies included glucocorticoids [36,66], radioactive iodine (^131^I) [40], and thyroidectomy [30,49]. Hypothyroidism was managed with levothyroxine replacement [7,14,23,26,42,46,48].

The majority of studies (*n* = 45) achieved clinical and biochemical remission within 1 to 4 months of initiating standard therapy. However, two fatal outcomes were reported: one case of myxedema coma in a patient with severe hypothyroidism and concurrent COVID-19 [33] and one case of fatal thyroid storm [62].

### 3.6. Risk of Bias and Quality of Evidence

The methodological quality of the included studies was appraised using the Joanna Briggs Institute (JBI) Critical Appraisal Tools.

The five analytical studies (cohort and case–control) were assessed as having a low-to-moderate risk of bias. Their strengths included clear participant selection, valid measurement of exposure (COVID-19) and outcome (AITD), and attempts were made to control for confounding factors such as disease severity and sex.

The 43 case reports and case series were well reported, with precise patient demographics, diagnostic details, treatments, and outcomes, and met the core JBI criteria for clarity. However, due to their inherent design limitations—including the lack of control groups, the potential for selective publication, and the inability to establish causality—they contribute to a body of evidence that has a high risk of bias when used to determine incidence or prove causation. The most frequent methodological limitation noted across these case-level studies was the inconsistent reporting of follow-up duration.

Consequently, the overall strength of evidence for a causal link between SARS-CoV-2 infection and AITD is low to very low under hierarchical evidence principles. It is built on numerous, geographically diverse, and inherently biased case-level reports, supported by a small number of more robust observational studies.

The significant heterogeneity in study designs and the predominance of low-level evidence precluded a meaningful quantitative meta-analysis. A narrative synthesis was therefore conducted, focusing on descriptive patterns of clinical presentation, laboratory findings, and outcomes.

The studies consistently suggested a temporal relationship between COVID-19 infection and the development or exacerbation of autoimmune thyroid disease. Graves’ disease was the most frequently reported phenotype and typically manifested within several weeks after SARS-CoV-2 infection. Hashimoto’s thyroiditis and mixed autoimmune forms were also observed within similar post-infectious intervals. The phenomena of immune transition (e.g., Hashimoto’s to Graves’ disease) have been reported and may indicate post-infectious immune reprogramming [68,69]. No meta-analysis or statistical pooling was performed because a substantial portion of the evidence consisted of case reports. Instead, the information was provided descriptively, based on clinical patterns, antibodies, and treatment responses. One of the referenced population-based cohort studies did not demonstrate a significant increase in new cases of autoimmune thyroid disease following COVID-19 infection, suggesting that the incidence of autoimmune sequelae is very low but may be an important marker of disease progression in individuals at risk [70].

Nevertheless, extensive searches of PubMed, Scopus, and Web of Science, as well as the inclusion of case reports across multiple geographic regions, helped mitigate the risk of selective reporting [71,72,73].

Overall, the results of the present review suggest that COVID-19 could be a precipitant of autoimmune thyroid diseases, especially Graves’ disease, several weeks after infection. The evidence supports immune-mediated processes, including dysregulated cytokines, molecular mimicry, and post-viral autoimmunity [74]. The results also indicate that thyroid function should be monitored in cases of nonspecific symptoms, such as fatigue, palpitations, or weight changes, after COVID-19 infection, even though most cases are resolved with standard therapy.

## 4. Discussion

This systematic review synthesizes evidence from case reports, case series, and five cohort studies published between January 2020 and December 2025. Our findings demonstrate a consistent temporal association between SARS-CoV-2 infection and the onset or exacerbation of autoimmune thyroid disease (AITD). Most frequently, this manifests as Graves’ disease (GD), followed by Hashimoto’s thyroiditis (HT) and mixed or transitional phenotypes. Studies also provided quantitative evidence of this hypothesis through the use of a cohort study. In COVID-19 patients, there was approximately a two-fold increase in the incidence of TPOAb compared to controls [22], and the levels of thyroid autoantibodies were found to be persistently elevated at six-month follow-up and were directly related to the severity of COVID-19 [23].

Additionally, post-COVID-19 GD exhibited both significantly higher fT3 values and a higher rate of progression toward thyrotoxicosis relative to non-viral-induced GD [25], suggesting that COVID-19 may induce or trigger AITD, particularly in those with moderate-to-severe infection. The findings from our systematic review of case reports, which detail the clinical trajectory of post-COVID-19 AITD, are strongly corroborated by large-scale epidemiological data. The population-based study by Tesch et al. provides robust, quantitative evidence of this association, demonstrating a ~40% increased relative risk for both Graves’ disease and Hashimoto’s thyroiditis after COVID-19, independent of traditional risk factors [73].

The thyroid gland may be particularly susceptible to SARS-CoV-2-induced autoimmunity due to its high expression of angiotensin-converting enzyme 2 (ACE2) and transmembrane serine protease 2 (TMPRSS2), which facilitate viral entry into thyrocytes [75,76]. Direct viral cytopathy, coupled with a robust inflammatory response, may precipitate thyroid dysfunction. Additionally, molecular mimicry has been demonstrated between SARS-CoV-2 spike protein epitopes and thyroid autoantigens—such as thyroid peroxidase (TPO), thyroglobulin (Tg), and the TSH receptor (TSHR)—suggesting that cross-reactive immune responses may contribute to the breakdown of self-tolerance [77]. Notably, the risk of AITD is consistent with a relationship to disease severity: mild or asymptomatic SARS-CoV-2 infection rarely triggers autoimmune thyroid dysfunction, whereas moderate-to-severe COVID-19, often accompanied by a pronounced cytokine response (e.g., elevated IL-6), is necessary to disrupt immune homeostasis. While our review highlights clinical manifestations, the work by Tesch et al. [73] adds weight to the autoimmune hypothesis by showing that the risk is not limited to a few case reports but is observable at a population level, with the highest risk seen in patients with severe cases of COVID-19, who often exhibit pronounced immune dysregulation [9].

This pattern of post-viral autoimmune triggering is not unique to SARS-CoV-2; other viruses, such as the Epstein–Barr virus, parvovirus B19, and hepatitis C virus, have been reported to induce thyroid autoimmunity through similar pathogenic pathways [68]. Furthermore, SARS-CoV-2 has been associated with a broad spectrum of autoimmune phenomena beyond the thyroid, including autoimmune hemolytic anemia, Guillain–Barré syndrome, and systemic lupus erythematosus, supporting the concept that the virus can provoke broad immune dysregulation via mechanisms such as molecular mimicry and bystander activation [69].

Consistent with the known epidemiology of AITDs, the reported cases showed a strong female predominance (~81%), underscoring the influence of sex hormones and immunogenetic factors in autoimmune susceptibility [78,79]. Clinically, post-COVID-19 GD typically presents with classic thyrotoxic symptoms—palpitations, tremors, and weight loss—alongside suppressed TSH, elevated free thyroid hormones, and positive TRAb. HT is characterized by elevated TSH, low fT4, and positive TPOAb/TgAb, often with corresponding sonographic findings. Notably, several reported cases exhibited overlapping or shifting phenotypes (e.g., conversion from HT to GD), underscoring the dynamic immune dysregulation following infection [32,35].

The majority of patients developed AITD within 2–8 weeks post-infection, consistent with a post-viral immune-mediated process. Standard therapies were generally effective, including antithyroid drugs (methimazole and propylthiouracil) for hyperthyroidism and levothyroxine for hypothyroidism. Most patients achieved remission within months. However, severe complications such as thyroid storm, myxedema coma, and rare extra-thyroidal manifestations (e.g., periodic paralysis, encephalopathy, and dilated cardiomyopathy) highlight the potential for significant morbidity and mortality, necessitating vigilant monitoring and multidisciplinary management [80,81].

Importantly, not all thyroid dysfunctions in COVID-19 survivors represent true autoimmune thyroid disease (AITD). Non-thyroidal illness syndrome (NTIS), characterized by low fT3 with low or normal TSH, is common in severe systemic inflammation and must be distinguished from AITD to avoid inappropriate immunomodulatory treatment [75]. Furthermore, while our review suggests a plausible causal link, population-based cohort data indicate that the absolute increase in AITD incidence after a COVID-19 infection may be modest, implying that SARS-CoV-2 likely acts as a trigger in genetically or immunologically predisposed individuals rather than causing de novo autoimmunity at a population level [5].

### 4.1. Clinical Implications and Diagnostic Challenges

The symptomatic overlap between thyrotoxicosis (fatigue, palpitations, and anxiety) and post-acute sequelae of SARS-CoV-2 (PASC or “Long COVID”) poses a significant diagnostic challenge. Elevated fT3/fT4 ratios observed in post-COVID-19 GD cases may reflect a more aggressive thyroidal response, warranting a high index of suspicion in at-risk patients. We recommend considering thyroid function testing (TSH, fT4, and fT3) and, when indicated, thyroid autoantibodies in individuals presenting with persistent or new-onset constitutional, cardiovascular, or neuropsychiatric symptoms weeks to months after COVID-19—especially in women and those with personal or family histories of autoimmunity.

### 4.2. Methodological Considerations in Cohort Studies

The five cohort studies included in this review varied in the rigor of their design and control group selection. The prospective study by Rossini et al. [22] used age- and sex-matched controls from the general population, strengthening its validity. In contrast, the retrospective cohort studies by Di et al. [23], Khazaal et al. [24], and Ostapchuk [26] employed designs that excluded such rigorous comparator groups, limiting the interpretability of between-group differences. Furthermore, the absence of baseline thyroid autoantibody screening in several cohorts resulted in the fact that incident autoimmune thyroid disease (AITD) in patients post-COVID-19 infection cannot be definitively distinguished from pre-existing undiagnosed conditions. While the case–control study by Gökçay Canpolat et al. [25] provides valuable data on the phenotype of post-COVID-19 Graves’ disease, its design does not allow for calculating incidence or relative risk. Future research should prioritize prospective cohort studies with baseline serological screening, well-matched controls, and longitudinal follow-up to more rigorously assess incidence and causality.

### 4.3. Limitations of the Evidence

The current body of evidence has several limitations. Most included studies are case reports or small series, lacking control groups with generalizability. Follow-up durations are often short, and reporting of laboratory and imaging data is inconsistent, complicating cross-study comparisons. Publication bias likely favors severe or atypical presentations over subclinical cases. Furthermore, the diagnostic criteria for AITD vary, and early pandemic studies sometimes conflate autoimmune thyroiditis with subacute or non-thyroidal illness.

Under typical evidence hierarchy systems such as GRADE, the preponderance of case-level information will place the total confidence in the evidence that a relationship exists between SARS-CoV-2 infection and AITD as “low” to “very low”. No studies included in this review were randomized controlled trials or large prospective cohorts with predetermined autoimmune endpoints. While these case reports are substantiated by a large body of literature from all over the world—thus corroborating Tesch et al.’s [73] findings of their cohort study, they provide somewhat supporting but still inconclusive evidence. The fact that they do suggest further research may have to be done prospectively and utilize the collection of pre-exposure blood samples at baseline would allow researchers to demonstrate an association due to cause-and-effect.

The absence of a mandatory autoantibody criterion introduces the risk of misclassification bias. Biochemical thyroid dysfunction following COVID-19 is not pathognomonic of autoimmune disease; subacute (de Quervain’s) thyroiditis, non-thyroidal illness syndrome, and direct viral thyroid injury can produce similar biochemical profiles. Without serological confirmation of autoimmunity, the true proportion of cases attributable to AITD versus other post-viral thyroid pathology cannot be determined with certainty. This represents a fundamental limitation of a review reliant on case-based literature, and limits the specificity of conclusions regarding autoimmune mechanisms. A further limitation is the potential confounding effect of delayed medical presentations during the pandemic period. Patients who deferred seeking care could have been affected by more advanced disease, which could account in part for the reportedly greater severity of thyrotoxicosis at diagnosis and the fatal outcomes described. This temporal confounder limits causal inference.

The absence of a mandatory autoantibody criterion in the eligibility criteria introduces the risk of misclassification bias. Biochemical thyroid dysfunction in the context of COVID-19 does not exclusively represent true autoimmune disease. Without serological confirmation, some included cases could represent non-thyroidal illness or viral thyroiditis rather than AITD.

The predominance of case reports in our review, while valuable for characterizing the disease spectrum, is susceptible to publication bias. However, the consistency of our findings with the results from the large, unselected cohort study by Tesch et al. [73] suggests that the observed association between SARS-CoV-2 and AITD is a genuine phenomenon rather than a result of selective reporting.

Our review process was limited by the exclusion of articles not written in the English language and reliance on three major databases. These constraints may introduce language and selection biases. The heterogeneity of available data precluded meta-analysis, and causal inferences should therefore be made with caution [82,83].

Because of the way we conducted this study through case reports, there is likely a type of ascertainment or detection bias for why we found such high frequencies of reported Graves’ disease. As opposed to prospective cohort studies that screen all patients equally, case report research typically presents clinically apparent cases of diseases. Therefore, what we see as more cases of Graves’ could possibly be the result of this ascertainment bias rather than the actual prevalence of post-COVID-19 autoimmune thyroid diseases.

### 4.4. Future Directions

Further support for persistence in risk is provided by a prospective 4.5-year observational study showing an increased association of SARS-CoV-2 with the development of new thyroid diseases after SARS-CoV-2 infections, suggesting that it may be a longer-term consequence of SARS-CoV-2 illness [84].

Prospective, multicenter cohort studies with long-term follow-up are needed to establish the true incidence, natural history, and risk factors for post-COVID-19 presentations of AITD. Research should focus on elucidating pathogenic mechanisms—including T-cell responses, cytokine profiles, and genetic susceptibility—to identify high-risk individuals and inform targeted monitoring strategies [85]. International registries and standardized reporting frameworks, in line with recommendations from organizations such as the World Association of Medical Editors (WAME) and the International Committee of Medical Journal Editors (ICMJE), can enhance data harmonization and deepen our understanding of the long-term endocrine consequences of SARS-CoV-2 infection [86].

## 5. Conclusions

The data currently available suggest that COVID-19 can cause an increase in the development of thyroid autoimmunity, especially Graves’ disease; however, these results are based on relatively few case reports. This development often occurs several weeks after a moderate-to-severe infection. Some cases describe more intense thyrotoxicosis and a higher complication rate, including thyroid storm and orbitopathy, though these observations cannot be generalized. Clinicians should consider assessing thyroid function and autoantibody status in patients with unexplained fatigue, palpitations, or changes in weight during the first several months post-COVID-19, especially if the patient is female or has a previous history of developing an autoimmune condition. Current evidence, however, is insufficient to support routine screening. Prospective studies with baseline serological sampling are needed to determine whether systematic thyroid monitoring in COVID-19 survivors is warranted and which subgroups would benefit most.

### Registration

PROSPERO registration number: CRD420261280025.

## Figures and Tables

**Table 1:** Comparative analysis of etiology, diagnosis, and outcomes of thyroid diseases associated with COVID-19.

No	Authors	Country	Type of Investigation	Diagnosis/Diagnostic Confidence	Time of Onset	Clinic-Laboratory Findings	Treatment	Outcome
1	Rossini A. et al. (2023) [22]	Italy	Prospective cohort study (*n* = 599)	Increased prevalence of AITD/high	3 months after hospitalization for COVID-19	15.7% of survivors were TPOAb-positive (vs. 7.7% in the control group). 95% of patients had euthyroid syndrome (normal TSH). US features of thyroiditis in 94.9% of Ab+ patients.	Observation (as most were euthyroid).	Significant increase in thyroid autoimmunity markers post-COVID-19 infection.
2	Jia Di et al. (2024) [23]	China	Retrospective multicenter cohort study	AITD: GD and HT/high	Baseline, 3, and 6 months follow-up post-COVID-19 infection	2339 patients. Increased levels of TPOAb and TgAb. A correlation was found between the severity of COVID-19 and thyroid-inflammatory derangements. High NLR (neutrophil-to-lymphocyte ratio) in the AITD group.	Standard protocols for GD and HT; management of COVID-19.	Dynamic recovery of thyroid function over 6 months, but autoimmune markers (antibodies) remained persistent in many cases.
3	Khazaal A.S. et al. (2025) [24]	Iraq	Retrospective cohort study	New-onset HT (Hashimoto’s)/high	Post-COVID-19 recovery (March 2021–Jan 2024)	780 patients screened. Elevated TPOAb/TGAb levels post-recovery. Linked to COVID-19 severity and female sex.	Standard thyroid hormone replacement (levothyroxine) where indicated.	Identification of COVID-19 as a trigger for persistent thyroid autoimmunity.
4	Gökçay Canpolat et al. (2025) [25]	Turkey (multicenter)	Multicenter case–control and retrospective cohort study	New-onset and recurrent GD/high	Median: 60 days (IQR: 30–120) after COVID-19 infection	79 patients (81% F, median age 42). Positive TRAb in 94.7% of cases. 16.5% had Graves’ orbitopathy.	Antithyroid drugs (mainly methimazole).	The majority achieved biochemical control; however, post-COVID-19 patients showed more severe thyrotoxicosis at presentation.
5	Ostapchuk V.A., (2023) [26]	Ukraine	Observational cohort study	Autoimmune thyroiditis relapse manifested with hypothyroidism/ high	2–6 months after moderate COVID-19	12 patients. Worsening thyroid structure on US (“Swiss cheese” → “honeycomb” pattern). Elevated TPOAb and TSH levels; 7/12 progressed to manifest hypothyroidism.	Levothyroxine dose increased by 25–50 μg/day to restore euthyroidism.	Most patients required higher replacement doses; hypothyroidism aggravated but stabilized with dose adjustment.
6	Edwards K et al. (2021) [27]	USA (UT Southwestern Medical Center, Dallas, TX)	Case series (2 patients)	New-onset GD with severe thyrotoxicosis (1 thyroid storm; 1 impending storm)/high	Concurrent or shortly after COVID-19 infection	Pt 1: 27 M, presented with thyroid storm (Burch–Wartofsky 55), TSH < 0.01, FT4 > 7.8 ng/dL, FT3 21.9 pg/mL, and TRAb 9.1 IU/L, TSI 8.43 IU/L; US—diffuse hypervascularity. Pt 2: 21 F, impending storm (Burch–Wartofsky 40), TSH < 0.01, FT4 > 7.8 ng/dL, FT3 15.4 pg/mL, and TSI 7.77 IU/L.	Pt 1: Esmolol + propranolol + methimazole + PTU + dexamethasone, iodine solution (SSKI). Pt 2: Methimazole+ propranolol + hydrocortisone.	Pt1: Full recovery (8 days). Pt 2: Marked improvement within 24 h; discharged stable.
7	Sawamura T. et al. (2024) [28]	Japan	Case report	New-onset GD/high	4 months post-COVID-19 resolved within 5 days, but new symptoms reappeared ~3 weeks later, and final diagnosis occurred at day 117 )	27F. Misdiagnosed as having Long COVID3 visits. Labs: TSH < 0.01, FT3 > 20 pg/mL, FT4 3.94 ng/dL, TRAb 5.9 IU/L, TgAb 1960 IU/mL, TPOAb 358 IU/mL. Goiter + increased uptake on 99mTc scan.	Thiamazole 15 mg/day (thionamide) with gradual dose reduction over time.	The thyroid hormone was normalized clinically and biochemically. Symptoms resolved. After 1 year of treatment, the dose was reduced to 5 mg every 2 days. No relapse reported.
8	Jiménez-Blanco S. et al. (2021) [29]	Spain	Case report (letter, 2 cases)	GD relapse/high	~1 and 2 months post-COVID-19 infection	Pt1(45F). TSH < 0.005, FT4 > 7.7, TRAb 28.7. US: hypervascular thyroid. Pt2 (61F)A TSH < 0.001, FT4 2.66, and TRAb 1.31; increased thyroid uptake.	Methimazole 40 mg/day → 5 mg/day; 10 mg/day.	Rapid clinical and biochemical improvement within ~3 months of treatment.
9	Lanzolla G et al. (2021) [30]	Italy	Case report	New-onset GD + Graves’ orbitopathy/high	2 months post-COVID-19 infection	33F developed overt hyperthyroidism with positive anti-TSH receptor antibodies and a diffuse hypoechoic thyroid pattern. Exophthalmos 21 mm, CAS 1/7).	Methimazole.	Thyroid function normalized; no relapse reported.
10	Montebello A et al. (2021) [31]	Malta	Case report	GD relapse/high	8 weeks post-COVID-19 infection	22F (prior Graves’ disease in remission). TSH 0.009 μIU/mL, FT4 38.8 pmol/L, and TRAb 6.9 IU/L were positive; ultrasound showed heterogeneous thyroid with increased Doppler flow.	Carbimazole 40 mg/day + propranolol 40 mg TID; then, the dose was tapered to 10–15 mg/day.	TSH and FT4 normalized within 2–3 months; continued on maintenance therapy.
11	Shinzato T. et al. (2024) [32]	Japan	Case report	New-onset GD/high	~1 month post-COVID-19 infection	40F. Tremors, palpitations, sweating, and weight loss. TRAb- and TPOAb-positive. US: diffuse enlargement and hypervascularity.	Methimazole + propranolol therapy initiated.	Clinical improvement after treatment with stabilization of thyroid hormone levels.
12	Dixit N.M. et al. (2020) [33]	USA	Case report	Myxedema coma (autoimmune hypothyroidism)/high	Concurrent with acute SARS-CoV-2 infection	69F., TSH 61.3 µU/mL, fT4 0.2 ng/dL, anti-TPO 33.4 IU/mL, and positive SARS-CoV-2 PCR; CT: pulmonary embolism and ground-glass opacities.	IV hydrocortisone 100 mg + IV levothyroxine 200 µg; ventilatory and vasopressor support.	Died on day 3 from multi-organ failure after cardiac arrest.
13	Du S. et al. (2024) [34]	China	Case series (5 pts)	Mixed: 3 Hashimoto → Graves; 1 Hashimoto relapse; 1 new painless autoimmune thyroiditis/high	2–8 weeks post-COVID-19	5 female patients developed or had transformed AITD after COVID-19; all had positive TPOAb and/or TRAb; hypothesized immune shift (Th1→Th2).	Methimazole or levothyroxine, depending on the phase.	All improved; 1 remission of GD and others stabilized.
14	Allam MM et al. (2021) [35]	Egypt	Case series (3 pts total; 2 autoimmune eligible)	Pt 1: GD relapse +GOPt 2: Hashimoto hypothyroidism relapse/high	Pt1: symptoms started ~May 2020 after COVID-19; Pt 2: deterioration in months after COVID-19	Pt 1: ↑ TRAb 11.2 and worsening ophthalmopathy; Pt 2: hormone requirement instability + progression to deeper hypothyroidism.	Pt1: carbimazole + prednisolone + selenium; Pt 2: levothyroxine with dose adjustment.	Rapid clinical/biochemical improvement; no ICU, discharged stable.
15	Pastor S. et al. (2020) [36]	Spain	Case report	GD relapse → thyrotoxic crisis/ Moderate	38 days post-COVID-19 (RT-PCR-positive)	45F (prior GD remission > 4 years) developed thyrotoxic storm (Burch–Wartofsky score 50). Labs: TSH < 0.01 μU/mL, and FT4 3.29 ng/dL. COVID-19 RT-PCR-positive. No antibody data reported.	Atenolol 50 mg q12h + hydrocortisone 100 mg q8h + thiamazole 30 mg load → 30 mg q6h.	Rapid clinical and biochemical improvement; no ICU required. Discharged stable.
16	Ghareebian H. et al. (2022) [37]	USA	Case report	New-onset GD/high	A few days after acute COVID-19 infection	48M. TSH < 0.010; Free T4 elevated (2.8 ng/dL). Initially misdiagnosed as viral thyroiditis → given steroids. US: heterogeneous thyroid with increased Doppler blood flow. Thyroid-stimulating immunoglobulin was positive (TSI 6.31), confirming GD.	Initially, prednisone → after autoimmune confirmation, switched to methimazole 10 mg BID + metoprolol succinate 25 mg daily.	Good clinical improvement; outpatient follow-up stable.
17	Harris A. et al. (2021) [38]	USA	Case report	New-onset GD/high	16 days post-COVID-19 infection	21F. TSH 0.01 mcIU/mL, Free T4 3.8 ng/dL. TRAb 17 IU/L, and TSI 2.6 index. Tachycardia, palpitations. Family history: mother with hypothyroidism.	Methimazole+ β-blockers.	Clinical and biochemical improvement.
18	Feghali K. et al. (2021) [39]	USA	Case series (3 pts total; 2 autoimmune eligible)	(1) New-onset HD and severe hypothyroidism (2) New-onset GD/high	6 weeks (Hashimoto) and 8 weeks (Graves) after COVID-19 resolution	Pt 1 Hashimoto: TSH 136, fT4 0.2, anti-TPO > 900, and anti-Tg > 1000; Pt 2 Graves: TSH < 0.01, fT4 2.1, T3 216, TSI 309, and uptake 47.1%	Pt1: Levothyroxine 112 µg/day; Pt 2: Methimazole 10 mg/day + propranolol.	Both improved over several weeks with therapy; clinical and biochemical improvement was documented.
19	Deng L (2024) [40]	China	Case report	New-onset apathetic Graves’ disease/high	10 days post-COVID-19 infection	60F. Presented with anorexia, vomiting, jaundice, and exhaustion. TSH < 0.005, FT3 13.6 pmol/L, FT4 50.5 pmol/L, TgAb 1047 IU/mL, TPOAb 151 IU/mL, and TRAb 2.91 IU/L. Marked liver injury (ALT 203, AST 372, TBIL 30.3 → later 93.9). CKD stage 4 baseline.	Methimazole (MMI) 10 → 20 mg (hepatotoxicity) → ^131^I; artificial liver support, hemodialysis.	Liver function normalized at 1 month. Thyroid function normalized at 4 months post-treatment.
20	Elhadd T. (2022) [41]	Qatar	Case series (10 pts total; 5 Graves cases eligible)	New-onset Graves’ disease (4) and GD relapse/high	Ranged 2 weeks → up to 8 months post-COVID-19	TRAb positive in all; one case progressed to thyroid storm after stopping ATD.	Carbimazole ± propranolol; β-blocker use.	All 5 GD cases achieved remission with treatment; TFTs normalized.
21	Tee LY et al. (2021) [42]	Singapore	Case report	New-onset HD/high	7 days after mild post-COVID-19 symptoms	45M. Severe fatigue and muscle weakness. TSH 6.49 µIU/mL, fT4 9.19 pmol/L, and anti-TPO > 2000 IU/mL → primary hypothyroidism. No neck pain or prior thyroid disease.	Levothyroxine 25 µg daily.	Clinical improvement within 5 weeks; partial normalization of TFTs.
22	Kim HE. et al. (2023) [43]	South Korea	Case report	New-onset GD/high	Positive for SARS-CoV-2 two days before presentation with thyroid storm	42F pregnant. (35 + 2 weeks of gestation) Seizures, altered mentation, and fever. TSH < 0.01, Free T4 1.86–1.95 ng/dL, T3 175 ng/dL, and TRAb 2.44 IU/L. US: diffuse heterogeneity + papillary carcinoma nodule.	Emergency cesarean section. Antithyroid therapy (propylthiouracil), glucocorticoids, beta-blockers, and supportive ICU care.	Consciousness returned, and her seizures resolved. Both mother and neonate survived.
23	Mateu-Salat M. et al. (2020) [44]	Spain	Case report	GD relapse (Pt1) New-onset GD (Pt2)/high	1–2 months after COVID-19 infection	Pt1 (60F) with prior GD remission since 25 y/o. New hyperthyroid symptoms (palpitations/fatigue), TSH suppressed, FT3 ↑, TRAb+, TPOAb+, and high iodine uptake. Pt2 (53F) Tremors/palpitations, TSH < 0.01, FT4 ↑ significantly, TRAb+, TPOAb+, TgAb+, and high iodine uptake.	Thiamazole + propranolol	Marked clinical and biochemical improvement (restored euthyroid state)
24	Trinh AN et al. (2023) [45]	Vietnam	Case report	New-onset GD/high	6–8 weeks after confirmed COVID-19 infection	28F. Palpitations, dyspnea, and fatigue. TSH < 0.01 mIU/L, fT4 significantly elevated (36.04 pmol/L), fT3 elevated (6.18 pmol/L), TRAb 31.7 IU/L, TPOAb > 1000 IU/mL, and TgAb 173 IU/mL. US; hypervascular thyroid + mild thyromegaly.	Methimazole 20 mg daily + β-blockers for symptom control	Symptoms resolved. TFTs normalized; stable at long-term follow-up.
25	Knack RS et al. (2021) [46]	Brazil	Case report	New-onset HT/high	20 days post-COVID-19	33F. Fatigue and severe hair loss. TSH 8 mIU/mL, fT4 0.5 ng/dL, anti-Tg 252 IU/mL, and anti-TPO 115 IU/mL. US: diffuse hypoechogenicity and heterogeneous parenchyma. Genetic polymorphisms in the TNF-α and IL-6 genes were detected.	Levothyroxine sodium (Levoid) 25 µg → 38 µg	Complete clinical and biochemical recovery within 4 months; antibodies normalized.
26	Mekni S. et al. (2025) [47]	Tunisia	Case report	New-onset Graves’ orbitopathy/high	~10 days after COVID-19 infection	48F. Prior history: toxic thyroid adenoma treated with RAI. Bilateral active moderate–severe GO. TRAb became positive (2.5 IU/L). MRI confirmed orbitopathy.	Levothyroxine 125 µg/day + IV methylprednisolone pulses; then oral prednisone ×3 months; and later orbital decompression surgery + strabismus surgery.	Symptoms improved significantly post-surgery; no complications.
27	Lee ZC et al. (2023) [48]	Singapore	Case report	New-onset HD	14 days post-COVID-19	33M. Generalized myalgia, elevated CK (5358 U/L), anosmia, taste loss, and respiratory symptoms during acute COVID-19 infection. Labs showed severe hypothyroidism (TSH 216 mIU/L, Free T4 < 5.50 pmol/L) and positive anti-TPO antibodies (78.53 IU/mL). EMG demonstrated a mild diffuse myopathic process.	Levothyroxine replacement was initiated with gradual dose titration to 150 µg/day.	Clinical improvement with decreasing CK levels and improved thyroid function.
28	Sebastian W.P. et al. (2024) [49]	USA	Case report	GD relapse/moderate	~3 weeks post-COVID-19	42F (known history of GD diagnosed ~6 months earlier; acute exacerbation (headache, bilateral eye pressure/proptosis, muscle weakness, palpitations, and fatigue). Free T4 5.57 ng/dL, Free T3 15.68 pg/mL, and TSH < 0.01 uIU/mL. Burch–Wartofsky score 40 (impending thyroid storm).	Propylthiouracil (PTU) + oral potassium iodide solution + propranolol; later curative thyroidectomy once euthyroid.	Improved, underwent thyroidectomy, and discharged 2 days post-surgery in stable condition.
29	Shermetaro A. et al. (2023) [50]	USA	Case report	New-onset GD/moderate	Not reported in abstract (stated as “post-COVID-19 infection”)	81M. Palpitations, tremors, dizziness, diarrhea, fatigue; atrial fibrillation with RVR; labs consistent with hyperthyroidism; Burch–Wartofsky score met criteria for thyroid storm; technetium thyroid scan: diffuse increased uptake compatible with GD.	Propylthiouracil (PTU), hydrocortisone, cholestyramine, and potassium.	Clinical improvement with resolution of symptoms and improved thyroid studies.
30	Ergun U. et al. (2025) [51]	Turkey	Case report	New-onset GD/moderate	1-month post-COVID-19	25M. Severe sweating, palpitations, diarrhea, weight loss (−15 kg), and tachycardia (HR 130). TSH 0.015 U/L, T4 4.78 ng/dL, and T3 24.78 ng/dL. US: heterogeneous parenchyma with increased vascularity.	Methimazole 40 mg daily + propranolol 40 mg BID.	Clinical improvement with medical treatment; follow-up TFTs improved.
31	Mohammed S.R. et al. (2021) [52]	Trinidad and Tobago	Case report	Relapse of GD/high	~9 days post-COVID-19 pneumonia	A 28-year-old female; previous Graves’ disease treated 5 years prior, euthyroid since 2019; after PCR-confirmed COVID-19 pneumonia, she developed palpitations/sweating/tremor beginning 9 days later; labs: FT4 46.2 pg/mL ↑, FT3 18.3 pg/mL ↑, and TSH 0.0 → hyperthyroid; TRAb elevated (3.2 mIU/mL) and TPOAb elevated (42.5 IU/mL) → confirms autoimmune disorder.	Carbimazole 15 mg b.i.d. + Atenolol 50 mg daily.	Symptoms resolved within 1 week, and the patient remained well at follow-up.
32	Shetty A.J. et al. (2025) [53]	India	Case report	New-onset GD/high	~1 month post-COVID-19	Young adult male. Developed acute thyrotoxic periodic paralysis after mild SARS-CoV-2 infection. Positive TRAb and anti-TPO antibodies; thyroid scintigraphy showed diffusely increased uptake; and suppressed TSH and elevated FT4.	Carbimazole + propranolol + potassium supplementation/correction.	Clinical improvement with stabilization of thyroid hormones.
33	De Giglio L et al. (2023) [54]	Italy	Case report	New-onset GD with simultaneous thyroid eye disease/high	~1 month post-COVID-19 infection	Tachycardia, tremors, and weight loss. Suppressed TSH, elevated FT4/FT3, and positive TRAb. Eye findings: ophthalmopathy consistent with thyroid eye disease.	Antithyroid drugs + management of ophthalmopathy.	Clinical improvement under therapy.
34	Milani N. et al. (2021) [55]	Iran (Mashhad University of Medical Sciences)	Case series (1 patient eligible)	GD relapse complicated by thyroid storm/high	Within 2–3 weeks post-COVID-19 infection	39M (known GD, non-compliant with methimazole) developed thyroid storm post-COVID-19 (TSH < 0.05 mIU/mL, ↑ FT4, agitation, AF, and fever 39 °C).	Methimazole, propranolol, hydrocortisone, iodine, remdesivir, and supportive care.	Case 1: complete recovery and discharge after 8 days.
35	Milic G. et al. (2024) [56]	Serbia	Case report	New-onset HT/high	~3 months post-COVID-19 pneumonia	53F. HT with positive anti-TPO and anti-Tg, euthyroid function, and severe autonomic dysfunction confirmed by ANS testing/HRV markers.	Prednisone + supplements (selenium, zinc, magnesium, vitamin D), beta-blocker (Concor), antioxidants (glutathione, coQ10), vitamins (B12, C, D), NADH, and collagen.	Thyroid function is stable after 3 years; antibodies remain elevated, but ANS function improves.
36	Urbanovych AM, et al. (2021) [57]	Ukraine	Case report	New-onset GD G/high	~3 weeks post-COVID-19	22F. Palpitations, tremors, anxiety, muscle weakness, sleep disturbance. Labs: TSH < 0.01 mU/L, FT4 ↑, FT3 ↑, TRAb (+).	Methimazole, methylprednisolone, and bisoprolol.	Gradual improvement; normalization of thyroid hormones within 3 months; dose reduction in all medications.
37	Boyle DC (2023) [58]	USA	Case report	New-onset GD high	First episode 4 weeks post-COVID-19; Graves’ antibodies became positive ≈ 12 weeks post-COVID-19	65F. First episode: subclinical hyperthyroidism, AF with RVR, TSH 0.004 mIU/L, regular Doppler US, and TSI/TRAb initially negative → presumed thyroiditis; methimazole stopped. Second episode 1 month later: overt thyrotoxicosis (TSH < 0.002, FT4 ↑, TT3 ↑) and delayed TRAb 2.50 IU/L, and TSI 0.58 IU/L, confirming GD.	Methimazole (15–20 mg/day), metoprolol, digoxin, and dofetilide; multiple cardioversions due to recurrent AF with RVR.	Return to normal sinus rhythm; stabilization of thyroid function on methimazole; clinical improvement.
38	Sousa B. et al. (2022) [59]	Portugal	Case report	New-onset GD/high	~3 weeks post-COVID-19	28M. Fatigue, dyspnea, palpitations, 8 kg weight loss, and diffuse goiter. TSH < 0.001 mU/mL, FT4 7.11 ng/dL, TT3 486 ng/dL. Autoimmunity: TRAb 8.1 IU/L (↑), anti-TPO 478 IU/mL (↑), and anti-Tg 352 IU/mL (↑). US: diffusely heterogeneous thyroid + nodular image. Scintigraphy: GD pattern; nodules excluded.	Methimazole 20 mg daily + propranolol 10 mg (symptom control).	After 1 month: improvement + subclinical hyperthyroidism. After 6 months: normalization of thyroid values, methimazole discontinued, and no relapse.
39	França et al. (2022) [60]	Portugal	Case Report (Clinical Letter)	New-onset GD/high	3 months post-COVID-19)	Persistent tachycardia (130–140 bpm), palpitations, fatigue, dizziness, tremors, and goiter. Suppressed TSH (<0.008 µUI/mL), elevated Free T4 (6.53 ng/dL) and Free T3 (>20.0 pg/mL), positive anti-TSH receptor antibodies (57 U/L), and negative anti-TPO/Tg antibodies.	Methimazole (titrated to 20 mg/day), later adjusted to a “block-and-replace” strategy with methimazole 5 mg/day and levothyroxine 50 µg/day. Beta-blocker (bisoprolol) initially used.	Significant improvement in symptoms with treatment. Course complicated by iatrogenic hypothyroidism; thyroid function stabilized after medication adjustment, with recovery of autonomy.
40	Hamouche et al. (2022) [61]	USA	Case report	New-onset GD/high	1 week post-COVID-19	Suppressed TSH, elevated fT4 and total T3, and positive TRAb and antithyroid antibodies.	Methimazole + beta-blocker	Symptoms resolved; thyroid function normalized with treatment
41	Hamzah F. et al. (2022) [62]	Indonesia	Case report	Thyroid crisis (storm) in a patient with pre-existing GD (previously in remission)/high	Concurrent with acute COVID-19 infection	31F, delirium, tachycardia (125 bpm), jaundice, and dyspnea; BWPS score 75; bilateral pneumonia on CXR. Labs: TSH 0.1 mIU/L, and FT4 7.26 ng/dL (ref 0.93–1.71); positive SARS-CoV-2 PCR (CT 18.23).	Propranolol, methimazole (60 mg), dexamethasone, Lugol’s solution, favipiravir, and supportive care.	Fatal (death within 6 h of admission due to multi-organ failure).
42	Khaja M. et al. (2022) [63]	USA	Case report	Hashimoto’s thyroiditis encephalopathy (steroid-responsive encephalopathy associated with autoimmune thyroiditis)/high	Acute phase (concurrent with COVID-19 infection)	34F, obese, presented with anxiety, behavioral changes, and involuntary head movements. Labs: Elevated TSH (8.04), Low T4 (0.6), Positive anti-microsomal antibodies. Positive SARS-CoV-2 RNA.	Levothyroxine (initial) and then high-dose IV steroids (methylprednisolone) due to encephalopathy.	Significant clinical improvement in neurological symptoms after steroid therapy.
43	Murakawa K. et al. (2024) [64]	Japan	Case report	Fulminant type 1 diabetes and autoimmune thyroid disease (AITD)/high	~1 week after SARS-CoV-2 infection	44F, presenting with diabetic ketoacidosis (DKA). Subclinical thyrotoxicosis: TSH 0.02 and FT4 1.63. Positive TPOAb and TgAb. High HbA1c (8.7%) despite a sudden onset.	Insulin for T1DM; monitoring of thyroid function for AITD.	Stabilization of glucose levels; persistent thyroid autoimmunity.
44	Nham E. et al. (2023) [65]	South Korea	Case report	Concurrent subacute thyroiditis (SAT) and Graves’ disease (GD)/high	1 month after COVID-19 infection	34F, neck pain, and palpitations. Labs: TSH < 0.01, FT4 4.58 (0.7–1.48), TPOAb (40.9), and TRAb (4.5). Initial SAT followed by GD.	Prednisolone (for SAT), then methimazole (for GD) and beta-blockers.	Resolution of SAT symptoms, but persistent hyperthyroidism requiring long-term antithyroid therapy for GD.
45	Pumphrey T. & Rizzo A. (2023) [66]	USA	Case report	Thyroid storm (unmasked by COVID-19 infection)/high	Concurrent with acute COVID-19	44F, severe tachycardia (160 bpm), fever (39.5 °C), and altered mental status. BWPS score 65. TSH < 0.01, FT4 > 7.7. Positive for SARS-CoV-2.	Methimazole, propranolol, hydrocortisone, and Lugol’s solution.	Rapid improvement within 48 h; discharged on oral antithyroid medications.
46	Achour S. et al. (2023) [67]	Tunisia	Case report	New-onset GD with dilated cardiomyopathy/high	4 months post-COVID-19	48F; abdominal distension, edema, atrial fibrillation, and exophthalmos. Labs: TSH 0.008, T4 12.8, TRAb 35 IU/L, and anti-TPO positive. Cardiac MRI: biventricular dilatation and LVEF 29%.	Carbimazole, beta-blockers (bisoprolol), diuretics, ACE inhibitors, and digoxin.	Improvement in cardiac function and stabilization of thyroid status.

Abbreviations: Symbols: ↑, elevated or increased value; →, transition, progression, or change to (e.g., dose adjustment, disease conversion, or temporal sequence), AITD, autoimmune thyroid disease; CK, creatine kinase; FT3, free triiodothyronine; FT4, free thyroxine; GD, Graves’ disease; GO, Graves’ orbitopathy; HT, Hashimoto’s thyroiditis; MMI, methimazole; PTU, propylthiouracil; RAI, radioactive iodine; SSKI, saturated solution of potassium iodide; TPOAb, thyroid peroxidase antibodies; TRAb, thyrotropin receptor antibodies.

## Data Availability

All information obtained from the included studies is summarized in the main text of this manuscript. The extracted data form and complete evidence in Table 1 are available from the corresponding author upon reasonable request.

## Supplementary Materials

The following supporting information can be downloaded at: https://www.mdpi.com/article/10.3390/ijerph23060689/s1, Table S1: PRISMA 2020 Checklist Autoimmune Thyroid Diseases after COVID-19 Infection: A Systematic Review of Clinical Manifestation and Outcomes.
